# Consensus Statements Among European Sleep Surgery Experts on Tongue, Hypopharynx, and Supraglottis Associated with Snoring and Obstructive Sleep Apnea: Part 1: Evaluation and Decision Making

**DOI:** 10.3390/jcm15010080

**Published:** 2025-12-22

**Authors:** Ewa Olszewska, Andrea De Vito, Peter Baptista, Matej Delakorda, Clemens Heiser, Ryan C. T. Cheong, Guillermo Plaza, Olivier Vanderveken, Nuria Pérez-Martin, Bhik Kotecha, Joachim T. Maurer, Claudio Vicini

**Affiliations:** 1 Department of Otolaryngology, Sleep Surgery Center, Medical University of Bialystok, 15-276 Bialystok, Poland; 2 Department of Surgery, Morgagni-Pierantoni Hospital, Health Local Agency of Romagna, 47121 Forli, Italy; dr.andrea.devito@gmail.com; 3 Departmento de Orl, Clinica Universidad da Navarra, 31008 Pamplona, Spain; peterbaptista@gmail.com; 4 Department of Otorhinolaryngology and Cervicofacial Surgery, General Hospital Celje, 3000 Celje, Slovenia; 5 Department of Otorhinolaryngology/Head and Neck Surgery, Klinikum Rechts der Isar, Technical University of Munich, 80333 München, Germany; 6 Faculty of Medicine and Health Sciences, University of Antwerp, 2000 Antwerp, Belgium; 7 Consultant ENT and Sleep Surgeon, University College London Hospitals, London WC1N 3BG, UK; ryan.cheong@nhs.net; 8 Department of Otorhinolaryngology, Hospital Universitario de Fuenlabrada, Universidad Rey Juan Carlos, 28042 Madrid, Spain; guillermo.plaza@salud.madrid.org; 9 Department of Otorhinolaryngology, Hospital Universitario Sanitas La Zarzuela, 28023 Madrid, Spain; 10 Department of Otorhinolaryngology, Head and Neck Surgery, Antwerp University Hospital, 2650 Antwerp, Belgium; 11 Departament of Otorhinolaryngology, Hospital Universitario Infantil Niño Jesus, 28009 Madrid, Spain; nperezm@salud.madrid.org; 12 Nuffield Health Brentwood, Brentwood CM15 8EH, UK; 13 Division of Sleep Medicine, Department of ORL-HNS, Medical Faculty Mannheim, University Heidelberg, 69117 Heidelberg, Germany; 14 Department of Information Technology, Technical University of Applied Sciences, 68163 Mannheim, Germany; 15 GVM Care & Research ENT Consultant, GVM Primus Medical Center, GVM San Pier Damiano Hospital, 48018 Faenza, Italy

**Keywords:** snoring, sleep apnea, obstructive sleep apnea, tongue, hypopharynx, supraglottis, consensus, diagnosis, decision making

## Abstract

**Introduction:** The tongue base, hypopharynx, and supraglottis (TngHpxSgl) play distinct roles in snoring and obstructive sleep apnea (OSA). **Aim of the Study:** To assess the level of consensus on the assessment and decision-making for the management of snoring and OSA associated with TngHpxSgl. **Methods:** A set of statements on the assessment and decision-making for the management of snoring and OSA associated with TngHpxSgl was developed based on the literature and circulated among 12-panel members of European experts on sleep surgery, using the modified Delphi method, seeking at least 80% consensus. Responses were categorized as agree or disagree for each statement, and the comments from the panelists were used to assess the level of consensus. Statements containing aggregated anonymized responses and comments were sent to each panel member in the second and final rounds of the survey. **Results:** The final set included 147 statements. Of these, 52.6%, 14.7%, and 5.8% achieved consensus among all 12, 11, and 10 panelists, respectively. **Conclusions:** There was a high level of consensus (73.2%) among European sleep surgery experts on the statements. This consensus will help establish standards and guide further research on snoring and OSA related to TngHpxSgl.

## 1. Introduction

Sleep-disordered breathing (SDB), including snoring and obstructive sleep apnea (OSA), involves multiple anatomical levels, with the tongue base, hypopharynx, and supraglottis (TngHpxSgl) contributing important roles [1,2]. While not commonly isolated sites of vibration and obstruction, TngHpxSgl play an essential role, either primary or secondary, at upper airway levels [3,4].

According to the S2k guideline of the German Society of Otorhinolaryngology, snoring is a sound produced during breathing while asleep due to the vibration of soft tissue structures in narrow regions of the upper airways. This narrowing is due to a sleep-associated decrease in the tone of the upper airway dilator muscles, which causes airflow to become turbulent and leads to vibration [5]. This definition maintains the core concept of muscle relaxation, airway narrowing, and tissue vibration, generally referring to soft tissue. The vibration of enlarged tissues within the oro- and hypopharynx that causes snoring sounds results in inflammation, which is a predisposing factor for developing OSA. A global meta-analysis estimated that more than 930 million adults aged 30–69 suffer from mild to severe OSA, and more than 420 million suffer from moderate to severe OSA. Up to 90% of individuals with OSA remain undiagnosed (80–90% undiagnosed cases of moderate to severe OSA, approximately 93% of undiagnosed women with moderate to severe OSA, and approximately 82% of undiagnosed men with moderate to severe OSA) [6]. Patients with suspected or untreated OSA are at an increased risk of developing cardiovascular diseases, such as hypertension, heart attack, cardiac arrhythmias, heart failure, stroke, metabolic dysregulation, diabetes mellitus, and depression [7,8,9,10,11,12,13]. They also suffer from sleep deprivation, which can lead to impaired concentration and dysmnesia during the day [14,15,16].

Snoring and OSA can be treated using different approaches [17,18]. Selecting an appropriate treatment for snoring and/or OSA is a complex process that considers multiple factors, including patient age, comorbidities, disease stage, severity, and the pheno- and endotypes of OSA. Additionally, the anatomical characteristics of the upper airway and palate phenotypes play crucial roles. Findings from supplementary diagnostic tools, such as drug-induced sleep endoscopy (DISE), craniofacial computed tomography, and dynamic sleep magnetic resonance imaging, can also be integrated into the decision-making process [19,20,21,22]. Non-surgical treatment options are commonly discussed with patients with SDB. These include lifestyle modifications such as weight loss and improved sleep hygiene, the use of oral appliances, and engagement in myofunctional therapy. Positive airway pressure (PAP) therapy remains the first-line treatment for moderate-to-severe OSA, guided by appropriate clinical indications, followed by individualized PAP titration [23,24,25]. However, approximately 50% of patients prescribed PAP therapy are unable to use it consistently because of poor tolerance. Many patients discontinue therapy within the first few days to weeks of use, often citing discomfort, mask-related problems, or a perceived lack of benefit [26,27]. If PAP therapy proves ineffective, other nonsurgical treatments can be considered for patients with SDB, such as mandibular advancement devices and myofunctional therapy. In this context, surgical interventions may also be considered. These procedures are typically categorized as either minimally invasive or invasive. A critical aspect of surgical planning is identifying appropriate candidates and determining a specific surgical approach based on well-defined criteria. Additionally, for patients selected for surgery, it is essential to address perioperative considerations and establish a comprehensive preoperative plan, including strategies for postoperative management [28,29].

Healthcare systems, institutions, and professionals worldwide differ significantly in terms of their knowledge, experience, practices, and standards. Despite these variations, both patients and providers seek to establish shared concepts and consistent standards of care for the disease. Although achieving universally accepted global standards remains challenging, reaching a common framework within a single continent is a more realistic and achievable objective.

A series of consensus statements has been recently published about the surgery of the soft palate, which plays a central role in surgical treatment options in selected SDB patients [19,30,31].

In this consensus work, a panel of specialists in otolaryngology whose daily work routine involves sleep medicine and sleep surgery was convened to address the surgical management of TngHpxSgl for snoring and OSA. The goal was to develop consensus statements related to clinical assessment, decision-making, and surgical treatments. The first part of this report is related to the statements on assessment and decision-making for the management of snoring and OSA associated with TngHpxSgl.

## 2. Materials and Methods

The initial step involved inviting European experts whose medical practices were primarily centered on sleep medicine and sleep surgery, with specific expertise in TngHpxSgl. A working group of 12 members from seven European countries was thus established. These experts were identified through discussions among the core members of the team. Previous work on the soft palate had already established a panel of eight members, and those with significant experience in TngHpxSgl formed the initial core. They were then asked to recommend additional candidates for the new panel. After evaluating and discussing the qualifications of these candidates, a twelve-member panel was finalized. This study adopted a structured methodological approach, incorporating the modified Delphi technique to rigorously assess consensus levels. Initially, the first author conducted a comprehensive literature review to generate an initial set of statements. This review identified a broad range of information with varying levels of supporting evidence. Due to the scarcity of studies specifically addressing much of this information, an expanded list of statements was created. These statements were systematically categorized into sections covering history, physical examination, advanced evaluation methods such as imaging and endoscopic assessments, decision-making processes utilizing all this information, surgical management of these anatomical sites, postoperative outcomes and monitoring, and potential complications. Subsequently, all members of the working group reviewed the proposed statements to decide whether to accept, modify, or remove them, ultimately finalizing them for use in the Delphi rounds.

The statements were divided into two major sections: 1. Evaluation and Decision-making regarding surgery on the TngHpxSgl for snoring and OSA, and 2. Surgical treatment of TngHpxSgl for snoring and OSA. The first section included the following subsections: (a) history, (b) physical examination, (c) Imaging and DISE, and (d) decision-making. An Excel spreadsheet was prepared with all statements and sent to all panelists for the first round of the Delphi technique. The panelists were asked to indicate their agreement or disagreement with each statement and encouraged to provide comments or suggest modifications, where appropriate. After all responses were collected, the data were aggregated, and the number and percentage of panelists who agreed or disagreed with each statement was calculated. Comments on each statement were recorded. Apart from the first author, all responses from the individual panelists were kept anonymous and confidential.

A second-round iteration was conducted in accordance with the modified Delphi methodology by sending the aggregate responses and comments to each panelist, targeting a predefined consensus threshold of 80%. In addition to the aggregate summary, each participant was provided with their own prior round responses and given the opportunity to revise their feedback or submit additional comments for the second round. Because the statements had been reviewed and edited by all panelists before the first Delphi round, only minor edits were made to some statements in subsequent rounds. Following the collection of second-round responses and feedback, a final (third) round of statement evaluation was conducted using a similar methodology, and all the panelists were also requested to particularly check the statements that they were among the minority, that is, among those one, two, or three panelists that had dissented from the majority, and if so, to comment on their reason for the dissent. The responses to the third round were distributed as the final outcome.

## 3. Results

A total of 380 statements were established to circulate among the panelists regarding the management of TngHpxSgl for snoring and OSA. 147 statements were related to the evaluation and decision-making regarding surgery, and they are the focus of this paper. Of these, 60 were standalone statements, and 87 were sub-statements under nine parent statements. The remaining 233 statements relating to surgical treatment, complications, and post-operative care, will be the scope for another paper.

Responses after the third and final Delphi round, >80% consensus was achieved on 115 of 147 statements (78.2%). Of these, all 12 panelists had a consensus on 83 statements (56.5%), 11 out of 12 panelists had a consensus on 22 statements (15%), and 10 out of 12 panelists had a consensus on 10 statements (6.8%). We want to point out that the agreement among experts increased as the Delphi rounds progressed. Thus, the initial Delphi round revealed >80% consensus on 98 of the 147 statements (66.7%). Of these, all 12 panelists reached a consensus on 52 statements, 11 of 12 panelists reached a consensus on 26 statements, and 10 of 12 panelists reached a consensus on 20 statements. The second round revealed >80% consensus on 106 of 147 statements (72.1%). Of these, all 12 panelists reached a consensus on 62 statements, 11 of 12 panelists reached a consensus on 24 statements, and 10 of 12 panelists reached a consensus on 20 statements.

Notably, most statements were agreed upon. However, for three statements, there was a consensus (consensus among 10 out of 12 panelists) on “disagreement” with the statement. More panelists disagreed than those who agreed on 17 statements; however, a consensus was achieved on only three.

The first group of statements was regarding the “history” of snoring and OSA associated with TngHpxSgl. Of the 27 statements in this group, eight were standalone, and 19 were sub-statements under two parent statements (Table 1). A consensus among at least 80% of panelists (10 out of 12) was achieved on 81.5% of the statements (22 out of 27). Breaking down the level of consensus, all 12 agreed on 12 (44.4%) statements, 11 of 12 panelists agreed on 6 (22.2%) statements, and 10 of 12 panelists agreed on 4 (14.8%) statements.

There were 67 statements related to “physical examination” on OSA associated with obstruction at the TngHpxSgl levels (Table 2). There were 19 standalone statements and 48 sub-statements under five parent statements. A consensus was achieved among at least 80% of the panelists on 74.6% of the statements (50 out of 67). The breakout of the level of consensus demonstrated the following: All 12 panelists had a consensus on 35 statements (52.2%), 11 panelists had a consensus on 10 (14.9%), and 10 panelists had a consensus on 5 (7.5%) statements.

The group of statements related to “imaging and DISE” for evaluation of snoring and OSA associated with obstruction at the levels of TngHpxSgl, there were 29 statements (Table 3). There were nine standalone statements and 20 sub-statements under two parent statements. A consensus was achieved among at least 80% of the panelists on 89.7% of the statements (26 of the 29). Among them, the distribution of the level of consensus was as follows: all 12 panelists had a consensus on 20 statements (60%), 11 panelists had a consensus on 6 (20.7%), and 10 panelists had a consensus on none (0%) of the statements.

There were 24 statements on “decision making” related to the treatment of snoring and OSA associated with obstruction at the levels of TngHpxSgl (Table 4). There were nine standalone statements and 20 sub-statements under two parent statements. A consensus was achieved among at least 80% of the panelists on 70.8% of the statements (17 of the 24). The level of consensus was as follows: All 12 panelists had a consensus on 16 statements (66.7%), 11 panelists had a consensus on none (0%), and 10 panelists had a consensus on one (4.2%) statement.

## 4. Discussion

Traditionally, the evaluation of snoring and OSA has focused on the oropharynx, particularly the tonsils and soft palate [32]. However, decades of experience, not limited to the failures in the management of oropharyngeal structures, have expanded our understanding of the sites of vibration contributing to snoring and the sites of obstruction leading to OSA, extending into levels beyond the oropharynx [33]. Nasal obstruction has always been one of the targets; however, it was not within the scope of this study. The tongue, tongue base, and other hypopharyngeal and supraglottic structures have long been considered to contribute to upper airway obstruction [33,34]. The role of anatomic sites at TngHpxSgl has been extensively investigated, both as an isolated or in conjunction with obstruction at the upper levels. Concurrent obstruction may be primary, independent of the upper-level obstruction, or secondary to the higher-level obstruction, leading to airflow limitation. The concurrent presence of primary and secondary obstruction at the TngHpxSgl levels, i.e., potentially persistent obstruction at these sites even after the treatment of upper-level obstruction- creates a significant therapeutic challenge. Obviously, for the use of PAP treatment, which potentially resolves obstructions at all levels (except the nose), identifying the presence and relative contribution of different sites may not be critical. However, an unstable epiglottis can also be the cause of PAP intolerance [35,36]. When therapeutic interventions target specific anatomical structures—particularly surgical procedures aimed at reducing or reshaping distinct tissues—it is essential to identify all potential sites of obstruction, their relative contribution, and their possible interactions. Distinguishing primary from secondary obstruction sites has direct implications for clinical decision-making, especially in the surgical management of snoring and OSA.

This study aimed to establish the level of consensus among a group of prominent European sleep surgeons regarding their understanding of evaluation and decision-making for the surgical treatment of obstruction at the levels of TngHpxSgl. While the actual approach to surgical treatment will be presented separately, this manuscript focuses on the evaluation and decision-making for the treatment of snoring and OSA associated with the anatomical sites TngHpxSgl.

As with surgical treatment, substantial variability exists across countries, regions, and clinicians in the assessment and management of snoring and OSA. Even within Europe, level of awareness, concern, and willingness to seek diagnostic evaluation differ considerably, as do the availability and accessibility of diagnostic resources [30,37]. As in other parts of the world, obstruction at TngHpxSgl levels often receives limited attention in evaluation and treatment [38]. Limited knowledge, experience, and confidence in addressing these sites contribute to their under recognition [39]. Therefore, it is essential to strengthen knowledge and practice standards regarding snoring and OSA at these levels [40,41,42,43,44] and can lead to several positive outcomes such as earlier and more accurate diagnosis, more appropriate and consistent treatment, more efficient use of healthcare resources avoiding unnecessary testing and reduction in associated complications.

When there is an intent to seek consensus among practitioners, choices are made with respect to their purpose. Including most, if not all, practitioners in a field of medicine would demonstrate the degree of variability and highlight the lack of consensus on most diagnostic and therapeutic issues. An effort that aims to set a standard or guide for practitioners at large would select prominent participants with established practices in the field, as well as relative national and international prominence.

Our current work began by establishing a group of experts who had achieved prominence in Europe and beyond in the surgical treatment of snoring and OSA, with a focus on the sites of TngHpxSgl. Previous work on establishing consensus statements on the assessment and management of snoring and OSA related to the palate had already brought together eight panelists. Reassessing the experience and contributions of the potential participants led to the establishment of a 12-member panel. Having already established the methodology and expertise among most of these members, a decision was made to use the same steps as in the previous work to develop consensus statements.

Compared with the previous work and the three resulting publications [19,30,31], there were a few differences in methodology, aside from increasing the number of panelists. First, the method of establishing the set of statements was somewhat different. In an earlier study, the first set of statements was drafted by the first author and circulated, edited, and finalized only among three panelists before the actual Delphi rounds. This resulted in considerable suggestions not only from the other five but also from all eight panelists for editing and revision during and after the first and second Delphi rounds. This resulted in considerable changes in the content and the number of statements. This also necessitated the addition of new Delphi rounds to reach a consensus on the final set of statements. To avoid this, before circulating the set of statements as per the Delphi method, the draft set prepared by the first authors was sent to all panelists for their review, comments, and edits. As a result, there were only minor edits on the set of statements related to TngHpxSgl during the Delphi rounds, and there were no changes in the number of statements in each round. Moreover, because of this, a level of consensus among the panelists was reached after only three Delphi rounds.

The second difference utilized during the Delphi rounds, compared to the first work on palatal surgery, was that when the majority of the panelists demonstrated a consensus on disagreement, unlike the prior work where we had changed the statement to show the negative emphasis, we just left the statement as is; however, we presented such statements with a note of “DISAGREE,” indicating that the consensus was on disagreement on that specific statement. This more accurately reflected the position of each panelist, since modifying a statement to emphasize “negative” potentially did not always imply a similar meaning.

The third difference we applied in this work, compared to the work on palatal surgery statements, was that, in the third and last round, we suggested that panelists also check whether they were in the minority when the other panelists reached a majority consensus on agree or disagree. They were also asked to indicate their reasons for dissent. This may have contributed to a greater focus on nuances and a higher level of consensus.

One final distinction to highlight is the consensus threshold, which is set at 80% or more for this current work. Although there is no definitive rule for the Delphi threshold, it is customary to select a threshold around 80% [45]. In previous publications, the threshold was set at 75%. In an 8-panelist project, 75% equated to 6 panelists and was considered optimal. In the current work, the inclusion of 12 panelists allowed for a threshold of over 80%, corresponding to consensus among 10 or more panel members.

### 4.1. History

Regarding the set of statements pertaining to medical history, it is noteworthy that—all but two—achieved an agreement level exceeding 80%. Our first statement was consistent with the comprehensive approach to medical history, evaluation, diagnostic testing, and treatment options for snoring and OSA outlined in the literature [1,46]. In cases of failed treatment, a thorough reassessment, with particular attention to the tongue base and hypopharynx, has been recommended [47]. While 11 out of 12 panelists agreed that there was a higher chance of obstruction at the TngHpxSgl levels after prior surgery, one panelist stated that such a general statement may not be accurate, as it depends on the type of prior surgery.

Among the experts, there is a broad consensus that positional OSA, improvement with mandibular advancement devices (MAD), history of neuromuscular diseases, or use of medications that affect neuromuscular function are elements in the history that increase the suspicion of obstruction at the tongue and/or tongue base levels. A high level of consensus (>80%) was reached for factors such as prior OSA surgery, prior tonsillectomy, high BMI, high AHI, and recent weight gain, whereas heavy alcohol use did not. The literature also supports these statements [48,49]. For the elements in history that increased the suspicion of obstruction at the hypopharynx and/or supraglottis, complete consensus was reached only for a prior history of laryngomalacia. The definition, evaluation, and management of surgical failure have been extensively addressed in a comprehensive expert consensus study [47]. In our study, a greater number of panelists disagreed on the contribution of nasal obstruction to the hypopharynx and supraglottis. Eleven of the twelve panelists agreed that a history of PAP intolerance due to choking was suggestive of obstruction at these levels. Panelists reached full consensus on the use of sleep questionnaires (e.g., ESS, STOP-Bang) for assessing snoring and OSA related to TngHpxSgl, although these tools are not considered sensitive or specific for these sites [50,51,52,53].

### 4.2. Physical Exam

Physical examination for evaluation of snoring and OSA related to the anatomical levels of TngHpxSgl encompasses elements relevant to all anatomical regions, including a comprehensive clinical assessment of the entire head and neck, the nasal airway, velopharynx, pharyngeal wall, tongue base, and epiglottis, as well as a detailed evaluation of craniofacial skeletal relationships [54]. Guidelines and reviews have supported this comprehensive physical and endoscopic examination [55]. Consistent with the literature, current statements emphasized with unanimous full consensus the need for assessment of protrusion of the tongue and mandible, as well as the need for grading the visualization of the oropharynx and epiglottis. In addition, there was a full expert agreement that rigid or flexible endoscopy was insufficient for the evaluation of TngHpxSgl surgery. Although most panelists agreed on the importance of assessing neck circumference, those who disagreed cited its limited correlation with OSA, inability to replace other diagnostic tools, and lack of impact on surgical decision-making. Although not reaching consensus, eight panelists disagreed with the need for tongue muscle tone with the digital manometer (IOPI). Consensus among all panelists identified several physical examination findings as consistent with obstruction at the TngHpxSgl levels, including a large tongue base, enlarged lingual tonsils, and characteristic epiglottic appearances (retro-positioned, flat or omega-shaped, and soft/floppy epiglottis). The literature also supports these statements [56,57].

Guided by the consensus obtained, office flexible nasopharyngoscopy during specific maneuvers was recommended: when breathing through the nose versus mouth, when protruding the tongue, when advancing the mandible, and when supine. However, the need for neck flexion or extension during a flexible exam was disagreed upon by most of the panelists. Mueller maneuver was not one of the recommendations, and there was a consensus on disagreeing with the association between obstruction at the levels of TngHpxSgl and the presence of greater than 75% collapse during the Mueller maneuver.

In accordance with established guidelines [55], the necessity of a sleep study was emphasized as an integral component of the diagnostic evaluation and consideration of surgery targeting on TngHpxSgl for snoring and/or OSA. In cases where more than two years elapsed since the previous sleep study, all panelists endorsed repeating the assessment, even in the absence of changes in the BMI or symptomatology. Furthermore, there was unanimous agreement regarding the strong association between obstruction at the TngHpxSgl level and the presence of severe and/or positional OSA.

### 4.3. Imaging and DISE

Panelists demonstrated substantial agreement that imaging studies such as cephalometry, CT, MRI, or ultrasound are not needed prior to the surgery on TngHpxSgl. Some studies recommended these imaging studies and considered them useful only in selected cases, such as skeletal abnormalities, planning skeletal surgery, or complex anatomy [58].

Consistent with earlier reports, the panelists found DISE to be essential prior to and contributory to the success of surgery on TngHpxSgl [59]. A repeat DISE was considered essential by all panelists before surgery on TngHpxSgl in patients with a history of prior sleep surgery. Several studies have supported this approach to identify persistent or new sites of obstruction, to plan addressing such sites, as well as whether non-anatomical factors (such as airway collapsibility, epiglottis instability, and lateral wall collapse) have become dominant following previous surgery [47,60].

Panelists exhibited variable agreement regarding the specific maneuvers to be utilized when performing DISE. Whilst all agreed on visualizing the TngHpxSgl and glottis during inspiration and expiration, when the mouth is open and closed, and the mandible is manually protruded, one panelist disagreed on the need for rotating the body 30° because of a lack of supporting evidence, and one panelist disagreed with the need for applying PAP during DISE due to a lack of precision. The use of gradual advancement of the nasal trumpet during DISE for assessing obstruction at the TngHpxSgl and glottis received seven votes. There were more panelists who disagreed (eight) with the need for flexion and extension of the head. However, head flexion, extension, and lateral head rotation have been suggested for better evaluation of posture-related obstruction and tailoring treatment strategies [61,62]. The jaw thrust maneuver has been widely used and recommended in the literature, not only for surgical planning but also for predicting the outcomes of mandibular advancement device use [63,64,65,66,67]. Although not included in the current set of statements, the literature has recommended both the use of the Selector Advance Mandibular (SAM) device, designed to identify suitable candidates for MAD during DISE [68], and the tongue pull maneuver as predictive tools for the outcomes of tongue base interventions and hypoglossal nerve stimulation [67].

There was unanimous agreement on employing DISE to assess several critical information including the role of secondary obstruction at TngHpxSgl, from obstruction at higher levels including the nose, tonsils, and lingual tonsils; pattern of collapse at hypopharynx; the primary versus secondary collapse at the epiglottis and arytenoids; in the presence of arytenoid collapse, the likely pattern or cause, as well as the degree of collapse. Specific attention to supraglottic anatomy has been recommended in the literature to predict treatment outcomes and modify surgical protocols [69,70].

### 4.4. Decision Making

There is strong support in the literature for the need for a thorough review of prior diagnostic findings and treatment outcomes for decision-making in surgery for TngHpxSgl [1,71]. Past compliance and experience with PAP therapy, as well as learning expectations, are considered essential elements of shared decision-making [72]. Panelists had full consensus on many of these general aspects of decision-making, as well as detailed documentation of all this prior to the surgery on TngHpxSgl. Although guidelines and reviews emphasize the need for treatments with oral appliance therapy, positional therapy, and myofunctional therapy, prior to TngHpxSgl surgery, the panelists did not reach a consensus supporting such a position, pointing out that surgery may represent the first-line management option [73,74,75,76]. Consensus was reached that myofunctional therapy based on muscle tone and a 3-month minimum duration is not required. For decision-making regarding surgery, the need to differentiate between primary and secondary obstruction at the sites of TngHpxSgl was fully supported by the panel. Similarly, when the TngHpxSgl obstruction was thought to be secondary to the upper levels, including due to large tonsils, there was a full consensus on addressing the obstruction at upper levels first, including tonsillectomy alone or with other upper-level surgery if required, before surgery on the TngHpxSgl. When the lingual tonsils were the cause of secondary obstruction at TngHpxSgl, there was a complete consensus to address this as the first stage if isolated, before any other surgery on TngHpxSgl. However, most panelists disagreed with addressing the lingual tonsils concurrently with other upper-level sites as the primary step before surgery for TngHpxSgl, as hypertrophy of the tongue base by itself is not an indication for surgery; intervention is warranted only if tongue base collapse is observed during DISE. Otherwise, when the obstruction at TngHpxSgl was primary, all panel members were in full agreement on targeting these sites. Similarly, when this causality was not clear, the panel left the choice of multilevel surgery to all circumstances, including patient preference or the surgeon’s discretion. Such complex and challenging issues regarding decision-making on TngHpxSgl surgery continue to report heterogenous approaches in the literature [1,71,74,77,78,79].

### 4.5. Summary

Consensus among experts was significantly stronger for areas of agreement than for areas of disagreement. Nevertheless, the latter statements were retained to highlight unresolved issues within the clinical and therapeutic dimensions of OSA, underscoring the need for continued research and dialog. This research should ideally be prospective and in a multi-center setting in the international context.

Anatomical sites of TngHpxSgl should be included in the comprehensive evaluation of patients with OSA. Failure to identify and address these regions may result not only in persistent symptoms but also in residual OSA after isolated oropharyngeal procedures. DISE has emerged as the cornerstone of preoperative assessment, providing dynamic visualization of upper airway collapse patterns that static imaging cannot capture. However, interobserver variability and the lack of standardized scoring systems remain challenges in optimizing DISE-guided surgical planning.

The authors believe that the statements in the current study on decision-making and perioperative considerations regarding the surgical management of SDB, including snoring and OSA, will aid clinicians, particularly sleep surgeons, in managing these patients. Continued discussion is expected to enhance standardization and stimulate further research in areas where consensus is lacking.

Future research should focus on multicenter, prospective trials with standardized definitions and protocols for assessment, testing, and decision-making to achieve successful outcomes of surgery for snoring and OSA, targeting the TngHpxSgl either as isolated procedures or as individualized, multilevel treatment algorithms tailored to patient anatomy, comorbidities, and treatment goals.

## 5. Limitations

This study has several limitations. Although the number of panelists was expanded compared with prior consensus work on palatal surgery, it remained relatively small. This study is limited by the relatively small panel size (12 experts), which, while typical for the Delphi method, may limit generalizability. Nonetheless, the multidisciplinary and international composition of the panel increases robustness. The process of selecting panelists was subjective, as there was no clear criterion or definition of what constitutes an “expert” or a specific level of expertise required in the management of snoring and OSA to guide their selection and invitation to the working group. This method may have unintentionally excluded other qualified experts in the field throughout Europe. The choice to involve European experts does not imply that these 12 individuals are the only authorities on the subject. The lack of objective criteria in the panelist selection process is a notable shortcoming of this manuscript.

The second limitation was the inability to hold an in-person or online meeting to discuss the statements, especially those that did not achieve full or high consensus. This challenge has intensified with the panel’s expansion to 12 members. However, we believe that the effective distribution of statements and correspondence during the development phase, before finalizing the statements for the actual Delphi rounds, along with sharing responses and comments and addressing dissenting positions and opinions in the third and final rounds, has compensated for this limitation.

## 6. Conclusions

The assessment and management of snoring and OSA, particularly due to obstruction at the anatomical sites of the TgHpxSgl, exhibit significant variability worldwide. While pharyngeal sites have traditionally received broader attention in the management of snoring and OSA, there has been growing recognition of and clinical effort directed toward addressing obstructions at these additional sites, including TngHpxSgl. Nevertheless, knowledge, clinical experience, and standardized approaches for diagnosing and treating obstruction at these levels remain significantly less developed compared to those targeting the pharyngeal region. This gap underscores the importance of gathering and disseminating expert opinions on the assessment and management of TngHpxSgl-related obstruction in snoring and OSA.

The present study, which investigates consensus among leading European experts in the field, demonstrates a relatively high degree of agreement in the assessment and clinical decision-making processes concerning snoring and OSA. Establishing areas of consensus is crucial—not only to delineate where expert agreement exists but also to illustrate the diversity of opinions, emphasize the significance of topics lacking consensus, and identify key priorities for future research. This study, which examines consensus among leading European experts in the field, demonstrates a relatively high level of agreement in the assessment and clinical decision-making process regarding snoring and OSA.

## Figures and Tables

**Table 1:** Statements on the history of snoring and OSA associated with obstruction at the level of TngHpxSgl.

Statements 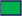 100% 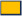 92% 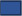 83%	% Consensus
1. Evaluation for OSA should be comprehensive, utilizing all standard assessment and diagnostic methods to identify all potential sites of upper airway obstruction. Both non-surgical and surgical treatment options should be considered, rather than focusing solely on the TngHpxSgl.	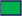 100
2. In patients with a history of failed non-surgical or surgical treatments for OSA, all potential sites of obstruction should be re-evaluated to guide appropriate surgical planning.	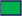 100
3. A comprehensive history is an essential part of the evaluation for surgery on TngHpxSgl.	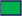 100
4. A comprehensive medical history is essential in evaluating for surgery on the TngHpxSgl. This should include an assessment of lifestyle factors (smoking, alcohol use), gastroesophageal reflux disease (GERD), and systemic comorbidities such as hypertension, diabetes, cardiac insufficiency, hypothyroidism, and neurologic disorders.	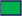 100
5. Other sleep disorders that are more relevant to the patients’ symptoms than OSA need to be ruled out to define the real necessity of OSA treatment.	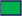 100
6. Evaluation for surgery on TngHpxSgl should include the history of the patient’s sleep habits (how many hours they sleep a day? Do they work shifts? Do they have insomnia?).	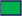 100
7. Ongoing complaints, symptoms, or suspicion despite the history of prior surgical treatment for OSA increase the possibility of obstruction at the sites of TngHpxSgl; however, the evaluation protocol should include all the general elements of an approach to failed surgery for snoring and/or OSA.	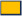 92
8. Elements in history that increase the suspicion of obstruction at the anatomical sites of the tongue and tongue base include:	
a. Prior surgical treatment for OSA	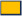 92
b. Prior tonsillectomy	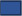 83
c. High body mass index (BMI > 35 kg/m^2^)	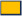 92
d. High apnea-hypopnea index (AHI ≥ 30)	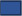 83
e. Recent weight gain	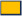 92
f. Heavy alcohol use	58
g. Improvement of OSA (but non-compliance) with Mandibular Advanced device (MAD)	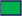 100
h. History of neuromuscular diseases	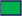 100
i. History of use of medications that affect neuromuscular function	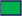 100
j. Positional OSA	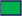 100
9. Elements in history that may increase the suspicion of obstruction at the hypopharynx and/or supraglottis include:	
a. Prior failed surgery on the soft palate and tongue and/or tongue base for OSA	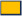 92
b. History suggesting the presence of lower airway obstruction, such as witnessed choking or gasping during sleep	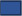 83
c. History of laryngeal symptoms like hoarseness or stridor	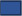 83
d. Patient mentioning that he/she feels something that blocks his/her airway during sleep	58
e. Severe GERD	67
f. PAP intolerance due to choking	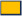 92
g. Previous history of laryngomalacia	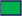 100
h. Improvement after myofunctional therapy	58
i. Symptoms of significant nasal obstruction—DISAGREE	58
10. Sleep Questionnaires such as ESS and STOP-Bang should be utilized as a part of the initial evaluation to quantify symptoms and screening for OSA severity.	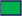 100

**Table 2:** Statements on physical examinations related to snoring and OSA associated with obstruction at the levels of TngHpxSgl.

Statements 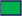 100% 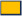 92% 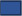 83%	% Consensus
1. A comprehensive clinical physical examination, including evaluation of craniofacial features and upper airway structures, is necessary in assessing patients for surgery on TngHpxSgl.	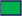 100
2. A comprehensive otorhinolaryngological clinical examination should be repeated after any prior treatment, prior to considering the surgery on TngHpxSgl.	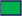 100
3. An office rigid endoscopy of the oropharynx and epiglottis is not sufficient for evaluation of TngHpxSgl surgery.	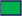 100
4. An office flexible endoscopy of the oropharynx and epiglottis is not sufficient for evaluation of TngHpxSgl surgery.	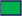 100
5. An office flexible nasolaryngoscopy of the oropharynx and epiglottis is necessary for evaluation of TngHpxSgl surgery.	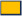 92
6. Assessment of mandibular position/occlusion and mandible protrusion is a necessary part of the physical examination.	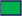 100
7. Assessment of the maxillary complex (width) is essential for evaluation of TngHpxSgl surgery.	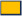 92
8. Assessment of tongue protrusion is an essential part of the physical examination for tongue surgery.	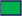 100
9. Grading of oropharyngeal visualization using standardized grading systems like the Modified Mallampati Score and Friedman Tongue Position is an essential part of the physical examination for TngHpxSgl surgery.	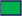 100
10. Grading of epiglottis visualization is an essential part of the physical examination for epiglottis surgery.	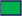 100
11. Assessment of neck size, specifically neck circumference, is essential in the physical examination for TngHpxSgl surgery.	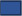 83
12. Measurement of tongue tone with a digital manometer is an essential part of the physical examination for TngHpxSgl surgery—DISAGREE	67
13. Elements in the physical examination that increase the suspicion of obstruction at the anatomical sites of TngHpx/Sgl include:	
a. Short neck	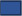 83
b. Large neck circumference (>43 cm in men, >41 cm in women)	75
c. Maxillary hypoplasia/retrognathia	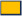 92
d. Mandibular micrognathia/retrognathia	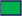 100
e. Surgically absent tonsils	75
f. Small tonsils—DISAGREE	67
g. Short soft palate/short and/or small uvula—DISAGREE	58
h. Large tongue base (FTP III and IV)	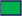 100
i. Enlarged lingual tonsils (grades 3 and 4)	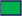 100
j. Inability to protrude the tongue	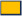 92
k. Retro-positioned, flat or omega-shaped, and soft/floppy and obstructing epiglottis/supraglottis	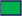 100
l. Presence of significant nasal obstruction—DISAGREE	58
m. Severe obesity affecting airway structures	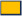 92
n. Low tongue tone with digital manometer	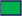 100
o. Limited mouth-opening—DISAGREE	75
p. Floppy and obstructing epiglottis/supraglottis	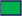 100
14. The presence of findings consistent with obstruction in the nose, nasopharynx, or oropharynx can be concurrent with obstruction at the tongue/tongue base and/or hypopharynx/supraglottis.	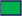 100
15. Office flexible nasolaryngoscopy should assess the following:	
a. Nasal obstruction on either nasal passage or the cause (septal deviation, nasal mass, polyp, turbinate hypertrophy, secretions).	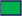 100
b. Nasopharyngeal obstruction and its cause (adenoid or lymphoid tissues, scarring from prior surgery).	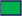 100
c. Assessment of retro-lingual space while breathing through the nose.	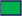 100
d. Assessment of change in the retro-lingual space dimensions.	
1. When breathing through the nose vs. the mouth.	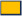 92
2. When protruding the tongue.	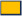 92
3. When advancing the mandible (slight advancement to maximum advancement).	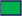 100
4. When being supine.	58
5. When neck flexion/extension—DISAGREE	75
e. Müller maneuver at the retro-lingual level.	58
f. Lingual-tonsil hypertrophy graded according to the Friedman scale.	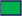 100
g. Shape of the epiglottis.	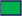 100
16. Findings in office flexible nasolaryngoscopy that are consistent with and/or suggestive (indicating) of obstruction at the tongue/tongue base include:	
a. Absence of nasal/nasopharyngeal obstruction—DISAGREE	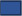 83
b. Absence of oropharyngeal obstruction (surgically absent or small tonsils)—DISAGREE	58
c. In the presence of nasal/nasopharyngeal and/or oropharyngeal obstruction, concurrent obstruction at the tongue base level.	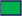 100
d. Presence of large lymphoid tissues at the tongue base (lingual tonsil).	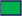 100
e. Inability to visualize vallecula.	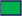 100
f. Inability to visualize the larynx (vocal cords).	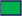 100
g. Limited improvement of the view of the larynx during vocalization.	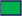 100
h. Flexible nasolaryngoscopy should include Muller’s Maneuver to assess for the potential degree of dynamic airway collapse.	50
i. Collapse greater than 75% in the retropalatal Muller maneuver—DISAGREE	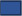 83
j. Retro-positioned, flat or omega-shaped, and soft epiglottis.	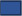 83
17. Findings in office flexible nasolaryngoscopy that are consistent with an/or suggestive (indicating) of obstruction at the hypopharynx/supraglottis include:	
a. Absence of nasal/nasopharyngeal obstruction—DISAGREE	67
b. Absence of oropharyngeal obstruction (surgically absent or small tonsils)—DISAGREE	67
c. In the presence of nasal/nasopharyngeal, oropharyngeal, and/or tongue/tongue. base obstruction, concurrent obstruction at the hypoglossus/supraglottis levels.	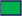 100
d. Presence of retroposition, flat or omega-shaped, and soft epiglottis.	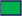 100
e. Presence of short aryepiglottic folds.	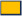 92
f. Presence of large, bulky, edematous arytenoid limiting visualization of the vocal cords.	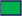 100
g. Limited improvement of the view of the vocal folds during vocalization.	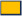 92
h. Indirect symptoms of reflux such as inter-arytenoid pachydermia.	50
18. A sleep study is essential for the diagnostic evaluation for TngHpxSgl surgery.	** 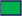 ** 100
19. Repeat sleep study is necessary after prior surgical treatment for OSA.	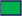 100
20. Repeat sleep study is indicated prior to surgical treatment for OSA if there is a long interval since the last sleep study or new symptoms suggestive of OSA progression.	
a. >1 year, even if there is no change in BMI/symptoms	58
b. >2 years even if there is no change in BMI/symptoms	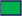 100
c. >10% increase in BMI	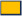 92
21. Sleep study criteria for surgical planning are not different for surgery TngHpxSgl.	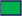 100
22. The presence of severe OSA should increase the suspicion of obstruction at the TngHpxSgl level, with or without other levels of obstruction.	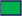 100
23. The presence and/or severity of positional OSA should increase the suspicion of obstruction at the TngHpxSgl.	** 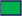 ** 100
24. The presence of low values of muscle tone obtained in digital manometry should increase the suspicion of obstruction at the tongue level, with or without other levels of obstruction.	67

**Table 3:** Statements on imaging and Drug-Induced Sleep Endoscopy (DISE) related to OSA associated with obstruction at the levels of TngHpxSgl.

Statements 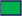 100% 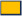 92% 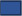 83%	% Consensus
**IMAGING**	
1. Cephalometry is not necessary prior to the TngHpxSgl surgery.	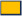 92
2. A CT scan is not necessary prior to the TngHpxSgl surgery.	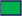 100
3. MRI is not necessary prior to the TngHpxSgl surgery.	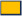 92
4. Ultrasound is not necessary prior to the TngHpxSgl surgery.	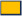 92
**DISE**	
1. DISE is essential prior to TngHpxSgl surgery.	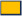 92
2. DISE improves the success rates of tongue surgery.	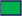 100
3. DISE improves the success rates of hypopharynx surgery.	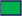 100
4. DISE improves the success rates of supraglottic surgery.	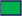 100
5. After a previously performed sleep surgery, a repeat DISE is necessary prior to new TngHpxSgl surgery.	** 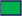 ** 100
6. During DISE, the following should be assessed using appropriate positioning and maneuvers: Use maneuvers to predict responsiveness to certain interventions (e.g., mandibular advancement devices). Degrees and collapse patterns (anteroposterior, lateral, concentric, latero-lateral) at each level should be documented.	
a. Evaluate the nasal passages, nasopharynx, soft palate, and uvula for their contributions to snoring and airway obstruction	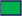 100
b. Concurrent residual soft palate collapse after pharyngoplasty	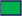 100
c. Assess the degree of obstruction at the TngHpxSgl during inspiration and expiration	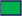 100
d. Change in the view of TngHpxSgl and glottis during inspiration and during expiration	
d.1. When the head is flexed—DISAGREE	67
d.2. When the head is extended—DISAGREE	67
d.3. When the body rotated (bed airplane rotation 30 degrees sideways) to the right/left	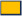 92
d.4. When the mouth is opened/closed	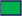 100
d.5. When the mandible is manually protruded (Jaw thrust maneuver)	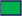 100
d.6. When (if possible) a nasal airway (nasal trumpet) is placed and advanced (a) past the choana, (b) past the lower level of soft palate, (c) past the tongue base (just above the epiglottis)	58
e. During DISE with a PAP degree of airway obstruction needs to be assessed at the oropharyngeal level and the levels of TngHpxSgl.	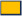 92
7. DISE for considering surgery on TngHpxSgl for OSA should try to assess/differentiate the following (if/when possible), utilizing the recommended positioning and maneuvers outlined above:	
a. Primary site of obstruction at the tongue/tongue base versus secondary to the nasal, nasopharyngeal/palatal/oropharyngeal obstruction.	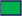 100
b. Role of tonsils (if present) on collapse of tongue base and hypopharynx, and the ability/inability to visualize the tongue base, hypopharynx, and supraglottis.	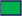 100
c. Role of a lingual tonsil (if present) on the collapse of the tongue base and the ability/inability to visualize the hypopharynx and supraglottis.	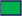 100
d. If present, a pattern of collapse at the hypopharynx: Anterior–posterior (AP) vs. Concentric vs. laterolateral (LL) vs. AP-LL.	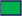 100
e. If the obstruction is present at the level of the epiglottis, it can be primary or secondary due to the presence of tongue collapse.	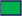 100
f. If there is primary epiglottic collapse, the type of epiglottic collapse needs to be differentiated (1. Anterior–Posterior (AP) collapse with a rigid component (trapdoor); 2. AP collapse with a floppy epiglottis and 3. Lateral-Lateral (LL) collapse with an omega-shaped epiglottis (book type).	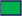 100
g. If obstruction is present at the level of the arytenoids and aryepiglottic folds, whether it is primary or secondary to the presence of upper-level collapse.	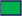 100
h. If obstruction is present at the level of the arytenoids, whether it is primary or secondary.	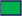 100
i. If there is secondary obstruction at the level of the arytenoids, whether it is secondary to 1. Short aryepiglottic folds, 2. Anterior–posterior collapse at the hypopharynx, 3. Circumferential collapse at the hypopharynx.	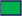 100
j. If there is primary arytenoid collapse, the severity of the arytenoid collapse needs to be graded.	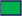 100

**Table 4:** Statements on decision-making related to OSA associated with obstruction at the levels of TngHpxSgl.

Statements 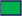 100% 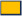 92% 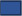 83%	% Consensus
1. Knowing the history of past diagnostic, treatment selections, and outcomes for snoring and OSA is important for the decision-making in TngHpxSgl surgery.	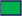 100
2. Learning the expectations of the patient regarding TngHpxSgl surgery is essential.	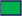 100
3. Knowing the level of compliance/intolerance for CPAP therapy is important in decision-making in TngHpxSgl surgery for OSA	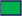 100
4. A shared decision-making approach should be adopted for TngHpxSgl surgery for OSA, ensuring patients are fully informed of risks, benefits, and alternatives, and their values and preferences are integrated into the treatment plan.	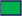 100
5. Before TngHpxSgl surgery for OSA, non-surgical treatment options (e.g., PAP therapy, myofunctional therapy, mandibular advancement device, positional therapy) should be recommended.	67
6. All patients should try improving their health quality measures (diet, weight control, exercise, and reducing alcohol) before TngHpxSgl surgery.	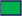 100
7. Those patients who are enthusiastic and willing to have a trial of improvement in their life quality to address the risk factors should repeat a sleep study if they achieve a stable condition before surgery on TngHpxSgl.	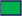 100
8. Before any surgical treatment, a PAP trial may be beneficial.	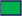 100
9. In a patient who is adequately informed of the options, the surgical risks, and without a pulmonary condition or any other severe co-morbidity, the patient’s desire not to try PAP treatment is sufficient to choose TngHpxSgl surgical options.	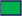 100
10. Patient reporting of non-compliance with PAP is not sufficient to choose TngHpxSgl surgical options.	58
11. Myofunctional therapy should be tried in patients with suspected/demonstrated hypotonic tongue and absence of BOT hypertrophy, before any surgery on TngHpxSgl for OSA.	67
12. Myofunctional therapy is recommended (if the genio-glossus muscle tone is <48 kPA in IOPI) before any surgery on TngHpxSgl for OSA—DISAGREE	75
13. Myofunctional therapy (if the genio-glossus muscle tone is <48 kPA in IOPI) should be tried at least 3 months before any surgery on TngHpxSgl for OSA—DISAGREE	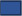 83
14. If indicated by DISE and there are no contraindications, the MAD should be tried before any TngHpxSgl surgery.	67
15. Before TngHpxSgl surgery, Positional Treatment should be tried in patients with positional OSA.	67
16. The presence of primary vs. secondary obstruction at the levels of TngHpxSgl needs to be differentiated.	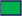 100
17. If the obstruction at the levels of TngHpxSgl is suspected to be caused by the upstream flow limitation or mouth opening at the levels of nose, nasopharynx, and/or pharynx, those sites need to be addressed surgically, prior to performing surgery at the levels of TngHpxSgl.	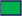 100
18. If the obstruction at the levels of TngHpxSgl appears to be primary, unrelated to any obstruction at the levels of nose, nasopharynx, and/or pharynx, surgery may be performed at the levels of TngHpxSgl only.	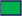 100
19. If it is not clear whether the obstruction at the levels of TngHpxSgl is primary or secondary to the Starling effect due to obstruction at the levels of the nose, nasopharynx, and/or pharynx, a surgeon will consider all other circumstances, including risk factors and a patient’s preference prior to performing surgery at the levels of TngHpxSgl.	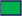 100
20. In the presence of large/obstructing tonsils (tonsil size 3 and 4), with other sites of obstruction at the nose/pharynx, tonsillectomy with surgery on those sites should be performed before surgery on TngHpxSgl for OSA.	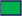 100
21. In the presence of large/obstructing tonsils, without other sites of obstruction at the nose/pharynx, tonsillectomy/pharyngoplasty should be performed before surgery on TngHpxSgl for OSA.	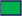 100
22. In the presence of a large/obstructing lingual tonsil (base of tongue lymphoid hypertrophy), with other sites of obstruction at the nose/pharynx, lingual tonsillectomy with surgery on those sites should be performed as the first stage, before surgery on other procedures on TngHpxSgl for OSA—DISAGREE	67
23. In the presence of a large/obstructing lingual tonsil (base of tongue lymphoid hypertrophy), without any obstruction at the nose or pharynx, lingual tonsillectomy should be performed at the first stage, before surgery on other procedures on TngHpxSgl for OSA.	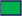 100
24. Detailed documentation of all elements of decision-making, patients’ expectations, and the information given by the surgeon is an essential part of decision-making for TngHpxSgl surgery due to snoring/OSA.	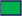 100

## Data Availability

Data is contained within the article.
